# Gender-Related Effects of Sex Steroids on Histamine Release and Fc*ε*RI Expression in Rat Peritoneal Mast Cells

**DOI:** 10.1155/2015/351829

**Published:** 2015-04-20

**Authors:** Samira Muñoz-Cruz, Yolanda Mendoza-Rodríguez, Karen E. Nava-Castro, Lilián Yepez-Mulia, Jorge Morales-Montor

**Affiliations:** ^1^Unidad de Investigación Médica en Enfermedades Infecciosas y Parasitarias, Instituto Mexicano del Seguro Social, 06720 México, DF, Mexico; ^2^Centro de Investigación Sobre Enfermedades Infecciosas, Instituto Nacional de Salud Pública, 62100 Cuernavaca, MOR, Mexico; ^3^Departamento de Inmunología, Instituto de Investigaciones Biomédicas, Universidad Nacional Autónoma de México, Apartado Postal 70228, 04510 México, DF, Mexico

## Abstract

Mast cells (MCs) are versatile effector and regulatory cells in various physiologic, immunologic, and pathologic processes. In addition to the well-characterized IgE/Fc*ε*RI-mediated degranulation, a variety of biological substances can induce MCs activation and release of their granule content. Sex steroids, mainly estradiol and progesterone, have been demonstrated to elicit MCs activation. Most published studies have been conducted on MCs lines or freshly isolated peritoneal and bone marrow-derived MC without addressing gender impact on MC response. Our goal was to investigate if the effect of estradiol, progesterone, testosterone, and dihydrotestosterone (DHT) on MCs may differ depending on whether female or male rats are used as MCs donors. Our results demonstrated that effect of sex steroids on MCs histamine release is dose- and gender-dependent and can be direct, synergistic, or inhibitory depending on whether hormones are used alone or to pretreat MCs followed by substance P-stimulation or upon IgE-mediated stimulation. In contrast, sex steroids did not have effect on the MC expression of the IgE high affinity receptor, Fc*ε*RI, no matter female or male rats were used. In conclusion, MCs degranulation is modulated by sex hormones in a gender-selective fashion, with MC from females being more susceptible than MC from males to the effects of sex steroids.

## 1. Introduction

Sex-based differences in infection and immunity suggest that sex steroids underlie these disparities [1]. Thus, in addition to the immune factors that regulate the complex immunoendocrine network, gender might have a significant function in shaping the immune response [2]. A reciprocal relationship between sex steroids and the immune system has been hypothesized for several years, and there is evidence that sex hormones influence the distribution and function of innate and adaptive immune cells [3]. In addition, gender and sex steroids govern the development and prevalence of many human diseases [4–6]. Thus, understanding the basis of differences in the immune response between genders is paramount for developing new approaches to prevent, diagnose, and treat infectious and autoimmune diseases.

Mast cells (MCs) are tissue-resident immune sentinel cells that are found in most vascularized tissues in close proximity to blood vessels, nerves, smooth muscle, and epithelial cells [7]. They are particularly abundant in sites that are exposed directly to the environment, such as the skin, airways, and the genitourinary and gastrointestinal tracts [7].

MCs immunological functions include the well-known IgE-mediated allergic responses, innate immune response against pathogenic infection, autoimmunity, wound healing, cardiovascular diseases, and cancer protection or promotion, among others. MCs can exert beneficial as well as detrimental effects through the release of potent inflammatory mediators, such as histamine, proteases, chemotactic factors, and cytokines [8]. MCs can be activated by a variety of stimulating factors, including IgE-specific antigens, complement components, neuropeptides such as somatostatin and substance P, cytokines, microbial products, and physical stimuli [9].

A number of* in vivo* and* in vitro* studies suggest that sexual hormones regulate MCs functionality and distribution in several tissues [10–14]. Strong data in the last years reinforced the idea that sex hormones have crucial effects on MCs behavior, not only in physiological conditions, but also in several pathological situations [15–18]. In this regard, a relationship between female hormones, MC-derived mediators and development of asthma and other allergic and inflammatory diseases has been suggested [4, 16, 19]. Furthermore, the presence of sex steroid receptors on MC indicates that sex hormones may exert their biological effects by binding to these receptors [20–23]. Interestingly, the expression of receptors, at least those for androgens, is different in MCs isolated from male or female subjects. Particularly, MCs isolated from human foreskin samples (male) have clearly higher levels than those from breast skin (female) and androgens modulate MCs effectors functions in a subset-specific fashion, being MCs from women more susceptible to testosterone effects [24], confirming that gender differences indeed are important when evaluating sex hormones effects.

The effect of sex hormones on MCs may be different depending on whether MCs are from male or female subjects. However, most of the studies analyzing the effect of sex hormones have been performed using MCs lines, freshly isolated peritoneal MCs from male rats or primary cultures of bone marrow-derived MCs, without addressing the influence of gender on MC response.

On the basis of these considerations, our goal was to investigate the effect of sex steroids, 17*β*-estradiol, progesterone, testosterone, and dihydrotestosterone, on MCs effector functions such as IgE-dependent and independent histamine secretion and MCs Fc*ε*RI expression, and correlate this effect to the gender origin of MCs donors (female or male) in rats.

## 2. Materials and Methods

### 2.1. Ethics Statement

Animal care and experimentation practices at the Instituto Mexicano del Seguro Social are constantly evaluated by the Institute Animal Care and Use Committee, adhering to the official Mexican regulations (NOM-062-ZOO-1999). Mexican regulations are in strict accordance with the recommendations in the Guide for the Care and Use of Laboratory Animals of the National Institute of Health (NIH) of the USA, to ensure compliance with established international regulations and guidelines. Efforts were made to minimize the animals suffering.

### 2.2. Animals

Adult male and female rats of the Sprague-Dawley strain aged 8 weeks were used in this study. All animals were appropriately housed in plastic boxes, in a light- and temperature-controlled room (12 h light : 12 h darkness; 22 ± 2°C) with sterilized food and water available ad libitum. All female animals from each experiment (3) were caged together to allow estrous cycle synchronization.

### 2.3. Isolation of Rat Peritoneal Mast Cell

Peritoneal mast cells (PMCs) were isolated as previously described [25]. Briefly, male and female Sprague-Dawley rats were euthanized using sodium pentobarbital anesthesia followed by cervical dislocation. Peritoneal cells were obtained by lavage of the peritoneal cavity using Hepes-buffered Tyrode's solution. PMCs were purified on a discontinuous percoll gradient. PMCs purity was >98% as determined by staining with toluidine blue. Cell viability was >99%. All protocols used for PMCs isolation from animals were approved by the Instituto Mexicano del Seguro Social.

### 2.4. Histamine Release Assay

For direct effect of sex hormones, PMCs suspensions (2.5 × 10^4^ cells) were incubated for 30 min at 37°C, with 17*β*-estradiol (E_2_), progesterone (P_4_), testosterone (T_4_), and dihydrotestosterone (DHT), at concentrations similar to the physiological values observed during rat estrous cycle, ranging from 10 picomolar (pM) to 10 nanomolar (nM), depending on the hormone. Pharmacological concentrations starting at 100 nM and over the same concentration were also tested. Supernatants and cell pellets were separated by centrifugation at 3000 g for 5 min at 4°C. Cell pellets were lysed and histamine content in supernatants and pellets was measured by a fluorometric method using* o*-phthalaldehyde. This assay is based on a phthalic condensation of histamine to yield a fluorescent product [26]. The fluorescent intensity was measured using a microplate fluorescent reader (Fluoroskan Ascent, Labsystems). Histamine release was expressed as percentage of the total cellular histamine content.

For IgE-dependent histamine release, PMCs (2.5 × 10^4^) were pretreated with each hormone for 90 min at 37°C and sensitized at the same time with antidinitrophenyl (DNP) IgE (10 *μ*g/mL). After washing away unbound IgE, PMCs were stimulated with DNP-HSA (100 ng/mL) for 30 min in the presence of the hormone. To test nonimmunological activation, PMCs were preincubated with sex hormones at 37°C for 90 min and then challenged with substance P (10 *μ*M) for 30 min in the presence of the hormone.

Histamine release expressed as percentage of the total cellular histamine content was calculated by the formula:

(1) %histamine  release =histamine  in  supernatanthistamine  in  supernatant  and  pellet×100.

### 2.5. Analysis of MCs Surface Fc*ε*RI Expression by Flow Cytometry

PMCs suspensions (5 × 10^4^ cells) were incubated with physiological and pharmacological doses of E_2_, P_4_, T_4_, and DHT for 16 h at 37°C. After washing, cells were incubated with Fc blocking reagent (CD16/CD32-Fc-gamma III/II Receptor) and incubated at 4°C with mouse monoclonal anti-Fc*ε*RI antibody (Abcam) in FACS buffer (PBS supplemented with 2% FBS and 0.02% sodium azide). Primary antibody was detected with FITC-coupled secondary antibody (Biolegend). For analysis of Fc*ε*RI expression in the presence of IgE, PMCs were incubated for 16 h at 37°C with rat myeloma IgE (Invitrogen) at 5 *μ*g/mL, in addition to each steroid or culture media alone. After washing, cells were incubated with FITC-mouse anti-rat IgE antibody (Thermo Scientific). Cells were finally washed with FACS buffer and analyzed by flow cytometry using a FACSAria (BD Biosciences) and data were analyzed with the FlowJo software. Relative Fc*ε*RI expression was calculated as follows: media fluorescence intensity (MFI). MFI = MFI hormone-stimulation/MFI unstimulated control. Mean values ± SEM are shown.

### 2.6. Statistical Analysis

The experimental design was a three-factorial experiment. Independent variables were (1) sex (male or female); (2) steroid used (E_2_, P_4_, T_4_, or DHT); or (3) stimulus (IgE-dependent or substance P). The dependent variables were histamine release and Fc*ε*RI expression on MCs. Statistical analysis of 2-way ANOVA and Bonferroni's test were performed with the software GraphPad Prism (version 5.0b for MacOSX, GraphPad Software, San Diego, California, USA (http://www.graphpad.com/)).

## 3. Results

### 3.1. Gender Differences in Sex Steroids Effect on Mast Cell Histamine Release

**Figure 1:**
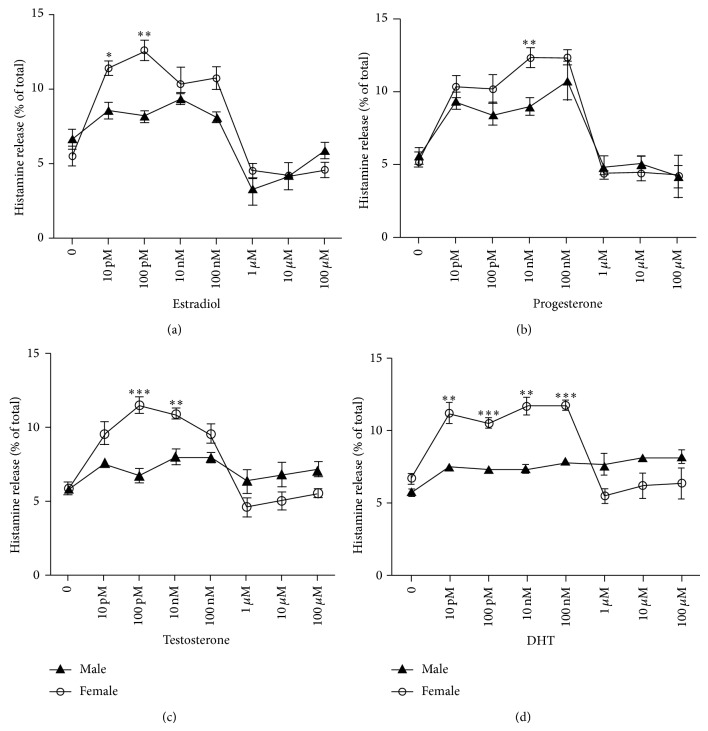
Gender and sex steroid concentration effect on histamine release in mast cells. Rat PMCs were treated with physiological (10 pM to 10 nM) and pharmacological concentrations (100 nM to 100 *μ*M) of estradiol, progesterone, testosterone, and DHT for 30 min at 37°C. Values are means ± SEM of three independent experiments in triplicate. ^*^ *P* < 0.05, ^**^ *P* < 0.01, and ^***^ *P* < 0.001 female* versus* male at each hormone concentration. Comparisons between genders were performed by one-way analysis of variance (ANOVA), followed by Bonferroni's multiple comparison test (GraphPad Prism). Basal histamine release was determined in nontreated PMC (no hormone treatment). Hormone concentration and gender had significant effect and the interaction is statistically significant (*P* < 0.001).

E_2_, P_4_, T_4_, and DHT at physiological concentrations caused histamine release (12 to 13.6%) in PMCs from female rats, significantly different (*P* ≤ 0.05) from the basal release (5 to 6%). In contrast, histamine release from PMCs from male rats was not significantly affected by any of the concentrations of the four hormones and remained similar to the basal release (Figure 1). There was a significant difference on sex steroids-induced histamine release between PMCs from male and female rats (*P* < 0.01). Furthermore, all the sex steroids tested caused the histamine release on PMC from female rats in a dose-dependent manner.

Significant histamine secretion, compared with the basal release of their own control group (*P* < 0.05), was observed when PMCs from female rats were treated with estradiol at concentrations of 10 and 100 pM, progesterone at 10 and 100 nM, and testosterone at 100 pM and 10 nM and in the case of DHT at concentrations ranging from 10 pM to 100 nM. Interestingly, pharmacological concentrations of the hormones, starting at 100 nM and over, did not have any significant effect on MCs histamine release, no matter whether testing was performed on PMC from male or female rats (Figure 1).

### 3.2. Gender Differences in Sex Steroids Effect on the Release of Histamine Induced by Substance P

**Figure 2:**
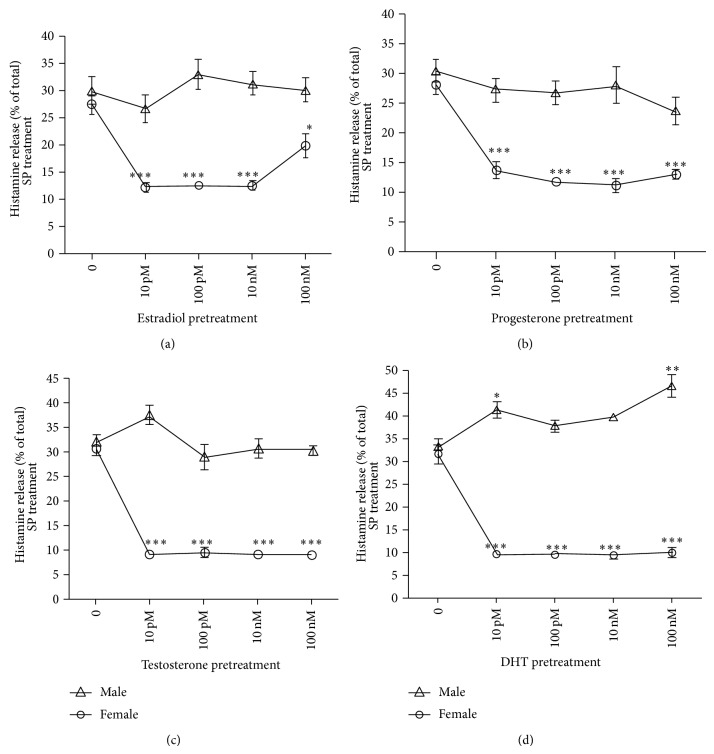
Effect of gender and sex steroid concentration on histamine release induced by substance P in mast cells. Rat PMCs were pretreated with E_2_, P_4_, T_4_, and DHT at 10 pM to 10 nM for 90 min at 37°C, followed by 30 min incubation with 10 *μ*M of substance P. Values are means ± SEM of three independent experiments in triplicate. ^*^ *P* < 0.05, ^**^ *P* < 0.01, and ^***^ *P* < 0.001 with and without hormone. Comparisons between genders were performed by one-way analysis of variance (ANOVA), followed by Bonferroni's multiple comparison test (GraphPad Prism). Hormone concentration and gender had significant effect and the interaction is statistically significant (*P* < 0.001).

The neuropeptide substance P was used to analyze the effect of sex hormones on histamine released induced by a classic MC secretagogue. Pretreatment of PMCs from female rats with E_2_, P_4_, T_4_, and DHT at physiological concentrations caused the inhibition of histamine release induced by substance P. This inhibitory effect was not observed in PMC from male rats (Figure 2). In PMCs from female rats, pretreatment with E_2_ starting at 10 pM up to 10 nM significantly inhibited the substance P-induced histamine release from 27 ± 3.2% (mean release induced by substance P alone) to 12.25 ± 1.3% (release induced by substance P after preincubation with the hormone at the indicated concentrations). P_4_ at all the concentrations used (from 10 pM to 100 nM) significantly inhibited the histamine release induced by substance P from 28.13 ± 2.3% to values ranging from 11.3 to 13.9%. A slightly inhibitory effect was observed when PMCs from male rats were pretreated with 100 nM P_4_; however, it was not significantly different from the same cells treated with substance P alone (Figure 2). Pretreatment with T_4_ and DTH also induced significant inhibition of histamine secretion, on PMCs from female rats, at all the concentrations used; in those cases, levels of histamine release induced by substance P were reduced about 10%. Interestingly, while pretreatment of PMCs from male rats with estradiol, progesterone, and testosterone did not affect the histamine release stimulated by substance P, pretreatment of the same cells with DHT potentiated the histamine release in a dose- and gender-dependent manner. Indeed, DHT pretreatment increased histamine secretion in the male rat PMCs significantly from 33.34 ± 3.4% to 41.55 ± 2.7 (10 pM DHT) and 46.8 ± 3.1 (100 nM DHT).

### 3.3. Differential Effect of Sex Steroids on IgE-Mediated Histamine Release in Peritoneal Mast Cells from Male and Female Rats

**Figure 3:**
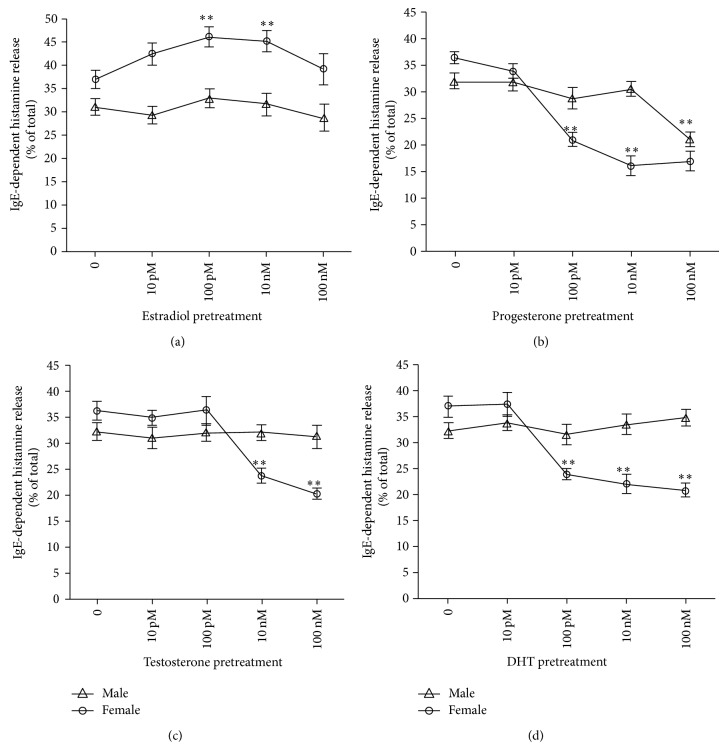
Differential effect of sex steroids on histamine release by PMCs from male and female rats, immunologically stimulated by the system IgE-DNP. PMCs were pretreated with each hormone at the indicated concentrations and sensitized at the same time with antidinitrophenyl (DNP) IgE (10 *μ*g/mL). PMCs were then stimulated with DNP-HSA (100 ng/mL) for 30 min in the presence of the corresponding hormone. The release of histamine was measured. ^*^ *P* < 0.05 IgE anti-DNP + DNP-HAS plus hormone* versus *IgE anti-DNP + DNP-HSA alone.

Incubation of IgE anti-DNP-sensitized PMCs with E_2_, P_4_, T_4_, and DHT at different physiological concentrations had different effect on the IgE-DNP mediated histamine release depending on the gender, type, and concentration of the hormone (Figure 3). Pretreatment of PMCs, obtained from female rats, with estradiol at 100 pM and 10 nM significantly increased IgE-DNP mediated histamine secretion. Nevertheless, estradiol pretreatment did not have any effect on the histamine secretion induced by IgE in PMCs from male rats.

In contrast, progesterone (100 pM to 100 nM) had a significant inhibitory effect on histamine secretion induced by IgE-DNP in PMCs from both male and female rats (Figure 3); however, the concentration of progesterone required (100 nM) to inhibit histamine release in PMC from male rats was about 1000 times higher than that needed to obtain the same effect in PMCs from female rats (100 pM).

Gender differential effects of testosterone and DHT on IgE-DNP-induced histamine in PMCs were also observed. While testosterone at concentrations of 10 nM and 100 nM was effective in reducing histamine release induced by IgE-DNP and DHT exert the same effect at concentrations of 100 pM, 10 nM, and 100 nM on PMCs from female rats, these hormones had no effect on IgE-DNP-induced histamine release in PMCs from male rats (Figure 3).

### 3.4. Effect of E_2_, P_4_, T_4_, and DHT on PMCs Basal Fc*ε*RI Expression

**Figure 4:**
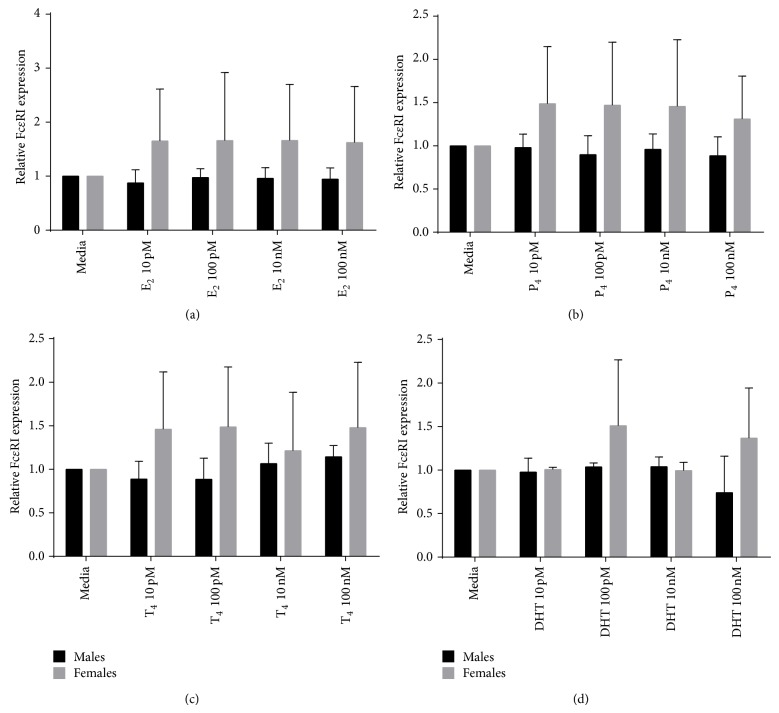
Fc*ε*RI expression on PMCs in response to E_2_, P_4_, T_4_, and DHT treatment. Fc*ε*RI relative expression in response to different concentrations of (a) E_2_, (b) P_4_, (c) T_4_, and (d) DHT. Relative expression was calculated as MFI (hormone-stimulated)/MFI (unstimulated control). *N* = 5 for males and *N* = 3 for females.

In addition to the effects of sex steroids on MCs histamine secretion, their effect on MCs surface expression levels of Fc*ε*RI was evaluated. The fluorescence intensity related to Fc*ε*RI expression observed in E_2_, P_4_, T_4_, or DHT-treated PMCs was the same with respect to untreated control PMCs, indicating no change on the surface expression (Figure 4). The control with only secondary antibody to look for nonspecific staining was not positive.

### 3.5. Effect of E_2_, P_4_, T_4_, and DHT on PMCs Fc*ε*RI Expression Stimulated by IgE

**Figure 5:**
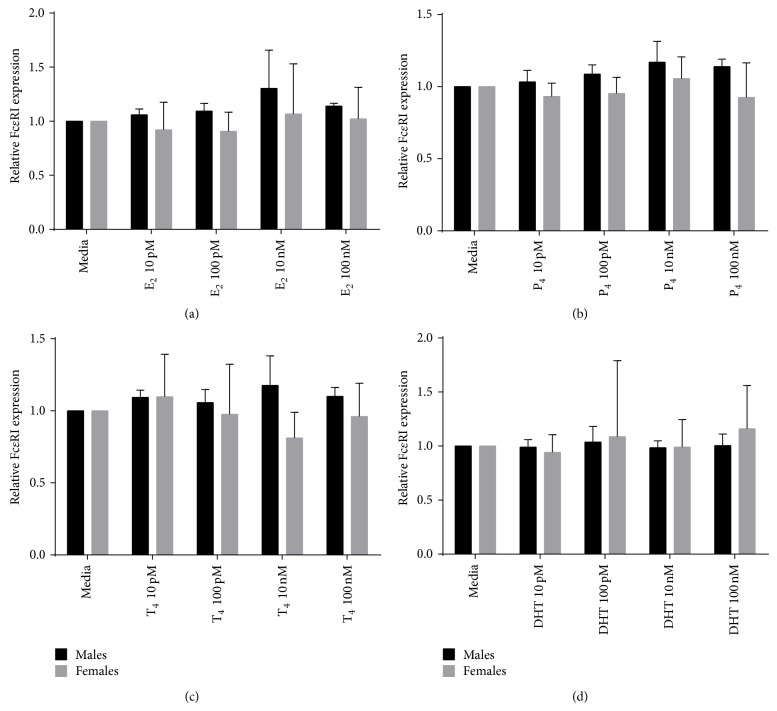
Fc*ε*RI expression induced by IgE stimulation in the presence of E_2_, P_4_, T_4_, and DHT. Fc*ε*RI relative expression induced by IgE stimulation in the presence of different concentrations of (a) E_2_, (b) P_4_, (c) T_4_, and (d) DHT. Relative expression was calculated as MFI (hormone-stimulated)/MFI (unstimulated control). *N* = 3 for males and *N* = 7 for females.

Finally, because MCs response to IgE-mediated stimulation can be influenced by regulation of Fc*ε*RI expression, the effect of each sex steroid on the upregulation of Fc*ε*RI expression induced by IgE was examined. As shown in Figure 5, there were no differences in the expression of Fc*ε*RI in MCs among males and females rats. Neither difference among treatments (E_2_, P_4_, T_4_, and DHT) was observed (Figure 5). Again, the control with only secondary antibody to look for nonspecific staining was not positive. Expression levels of Fc*ε*RI were modestly but evidently increased by incubation with IgE alone (data not shown).

The results of these experiments showed that in contrast to the ability to influence MC histamine release, sex hormones had no impact on MC surface expression of Fc*ε*RI, no matter whether female or male rats were used.

## 4. Discussion

Sex hormones regulate immunity and a well-documented dichotomy exists in the immune response between the sexes [2, 5, 6, 19]. Both clinical and animal models have demonstrated that the sex hormones, estradiol, progesterone, and testosterone, can mediate many of the sex-based differences in immune responses, in the susceptibility to infectious diseases and in the prevalence of autoimmune diseases [5, 6].

It is well known that sex steroids regulate the immune response by affecting immune system cells numbers, location, and function. A sex-associated difference in the number of MCs has been already described in a variety of tissues from rodents [10, 27]. Also, gender differences in MC reactivity and histamine content have been reported [28], indicating that sex steroids influence MC development and functionality; however, the exact mechanisms remain to be elucidated. The role of female hormones, estradiol and progesterone, on MC behavior have been the most intensely studied, while very few recent studies have addressed the role of androgens [24, 29]; besides, majority of these studies have been done using MCs lines or freshly isolated rat PMCs or mouse bone marrow-derived MC obtained from either female or male animals, without taking into account the influence of gender in the MC response.

In this study, PMCs isolated from both male and female rats were used to test whether sex hormones can influence, either directly or indirectly, the MC histamine secretion and MC phenotypic characteristics such as Fc*ε*RI expression, in a gender and concentration dependent fashion. The evaluation of the intrinsic capacity of sex steroids to induce in MCs, from female and male rats, the release of the preformed mediator histamine, was done at 30 minutes, because it is rapidly released upon MC activation. As transcription is not required for immediately histamine release, the effect of steroids relays only on nongenomic mechanisms and the expression of membrane cell activation receptors might be a key step in the signaling pathways leading to MCs degranulation after stimulation with sex steroids. We found that estradiol, progesterone, testosterone, and DHT at physiologic concentrations induce significant histamine release in MC from female rats but not in PMC from male rats. Interestingly, pharmacological concentrations of the sex steroids had no effect on MC secretion, in MC from neither male rats nor female rats. The latter observation is consistent with early results in which pharmacological doses of estradiol (1–100 *μ*M) have no direct effect on histamine release in PMC from male rats [11]. Other authors have shown that estradiol at concentrations between 1 nM to 10 *μ*M stimulated histamine release in PMCs from male rats; however, the level of release was not significantly above the basal release (5–10%) [30].

One possible explanation to the gender-specific effects of sex steroids in PMCs is that MCs isolated from female rats express more receptors for female hormones than those MCs from male rats, which make them more responsive to stimulation by estradiol and progesterone. In addition, structural variants of both estradiol and progesterone receptor exist, and the same steroid can produce opposite effects depending on the binding to one or another variant of its receptor [31, 32]. Sexual dimorphism in sex steroids receptors variants by MCs could result in varied effects by female hormones; however, comparisons in MCs receptor profiles between genders are lacking. Also, the precise role of sex steroid receptors variants in the steroid effects on MCs requires to be studied.

Regarding the gender-related effects of androgens the explanation could be different. Based on the results of a previous work showing that although human foreskin-derived MC (male) express higher levels of androgen receptor (AR) than breast skin-derived MC (female) the latter are more susceptible to testosterone [24]; the possibility exists that androgens are able to interact with nonsteroid receptors differentially expressed on the membrane of male and female rat MCs, which could possibly contribute to the male-female dissimilarity in MCs response to androgens.

Another possible mechanism to explain MC sexually dimorphic response to steroids is that sex steroids are activating MC intracellular signaling pathways in a sex-dependent fashion. This hypothesis is supported by previous work reporting that nongenomic actions of estradiol show sex differences in the activation of intracellular mechanisms in the mouse brain [33]. Moreover, sex-related differences in MAPK signaling pathway activation by estradiol were reported in male and female rat astrocytes [34]. Further studies are needed to explore these possibilities in order to demonstrate sex-specific mechanisms of response to sex steroids in MCs.

In contrast to the stimulating direct effects of sex hormones in female MC, when sex hormones were used as pretreatment of MC before stimulation with the neuropeptide substance P, all the hormones had a significantly inhibitory effect on histamine release in MC from female rats, being the effect of androgens highly inhibitory at all the concentrations tested. On the other hand, pretreatment of MCs from male rats with estradiol, progesterone, and testosterone did not have effect on substance P-induced histamine release. But, pretreatment of the same cells with DHT (10 pM and 100 nM) significantly increased substance P-induced histamine release, in contrast to the lack of effect observed when DHT was used alone. Since MCs were pretreated with sex steroids for 90 min before and during SP challenge for 30 min, the sex steroids could be affecting the SP-induced histamine release by a posttranscriptional pathway (may be affecting SP receptor expression and function) as well as by interfering with SP signaling pathways.

It was important to test the effect of sex hormones on MC degranulation induced by substance P, because, in addition to be present in cells belonging to the nervous system, substance P is also present in many other cells, such as immune cells, that are in close communication with MCs; furthermore, it is ubiquitous in human body and MCs and substance P together regulate many physiological and pathological processes [9]. Our data indicate that MCs from females in the presence of sex hormones do not respond any more to the regulation exerted by substance P. Better understanding of these two processes and how sex hormones exert both stimulatory and suppressive actions on MCs could lead to the development of inhibitors of the release of specific mediators with novel therapeutic applications.

Besides, sex hormones have also differential effects on IgE-mediated histamine release in PMCs from male and female rats. Treatment with estradiol of PMCs passively sensitized with IgE anti-DNP and challenged with DNP antigen significantly enhanced the IgE-mediated histamine release only in PMCs from female rats (at hormone concentration of 100 pM and 10 nM). Similar additive effects were observed when IgE-DNP-sensitized RBL-2H3 cells were treated with estradiol at same doses [23]. In contrast, it was reported that estradiol has no effect on IgE-mediated MC secretion, regardless of whether MCs from female or male rats were used; however, these results were derived using high concentrations (pharmacological) of estradiol [11].

For progesterone, an inhibitory effect was shown on histamine release from IgE-DNP-sensitized MCs from both male (at hormone concentration of 100 nM) and female rats (at 100 pM, 10 and 100 nM). This is consistent with previous studies reporting that progesterone (100 nM) inhibits histamine secretion from PMCs purified from male rats stimulated immunologically [14].

The differential effects of female hormones estradiol and progesterone on MC secretion have also been previously shown in ovariectomized-allergic rats, where estradiol but not progesterone significantly increased MC degranulation [16].

Regarding the effect of androgens on IgE-dependent MCs degranulation, both testosterone and DHT significantly inhibited histamine release induced by IgE-DNP on PMCs from female but not from male rats. So far, only two studies have recently analyzed the effects of androgens on MCs degranulation and some other MCs properties [24, 29]. The first one showed that treatment of the MC line HMC-1 with testosterone alone (100 nM), or in combination with different neuromuscular blocking agents, did not affect MCs degranulation. While the second one showed that treatment of MCs, derived from male foreskin or from female breast skin, with DHT at pharmacological concentration (400 nM) had no impact on IgE-mediated MCs degranulation as well as on other important properties of the MC such as the expression of Fc*ε*RI, c-Kit, or MC tryptase, in either MCs subset. Interestingly, DHT reduced slightly the level of the MC protease chymase and also inhibited significantly IL-6 production only in MC from female, whereas male MCs were resistant, even though the latter express higher androgen receptor levels [24].

Finally, we showed that estradiol, progesterone, and testosterone at physiological concentrations had no effect on Fc*ε*RI expression on the surface of PMC, from either male or female rats in both the presence and absence of IgE (a known upregulator of the Fc*ε*RI expression). This data is in agreement with previously reported results showing that the sex hormones, estradiol, progesterone, and testosterone, did not affect Fc*ε*RI expression on bone marrow-derived MC in either the presence or absence of IgE, whereas the glucocorticoids, dexamethasone, methylprednisolone, and hydrocortisone, were able to suppress the baseline levels of Fc*ε*RI expression as well as the upregulation of Fc*ε*RI induced by the presence of IgE [35].

It is worth noticing that our results indicated that gender and hormone concentration are important factors accounting for the direct effects on MCs histamine secretion. It is also clear that sex steroids affect function of MCs, by a yet unknown mechanism. Thus, starting to unravel the mechanism by which sex hormones regulate MCs responses would help to understand many MC-related pathophysiological alterations such as asthma and other allergic and inflammatory diseases that have a different prevalence in females than in males [4, 5].

## 5. Conclusion

Data obtained in the present study supports the evidence that effects of sex hormones, estradiol, progesterone, testosterone, and DHT, on MCs histamine release are dose- and gender-dependent. Furthermore, sex hormones can differentially modulate MCs degranulation, depending on whether they are used alone, as pretreatment followed by stimulation with the neuropeptide substance P or in conjunction with an immunologic stimulus when MCs are IgE-sensitized. In addition, MCs from female rats are more susceptible, in comparison to those cells from male rats, to the effects of sex hormones.

Sex hormones are among the many factors that contribute to disparate different immune responses in males and females. Indeed a strong reciprocal relationship exists between hormones and the immune response; therefore, demonstration that responses of MCs from female subjects differ from male subjects is crucial to address sex-based differences in a broad spectrum of physiologic, immunologic, and pathologic processes.
